# PGM3 insufficiency: a glycosylation disorder causing a notable T cell defect

**DOI:** 10.3389/fimmu.2024.1500381

**Published:** 2024-12-24

**Authors:** Linlin Yang, Barbara Zerbato, Alex Pessina, Luca Brambilla, Virginia Andreani, Stefanie Frey-Jakobs, Manfred Fliegauf, Mohamed-Ridha Barbouche, Qiaoxia Zhang, Ferdinando Chiaradonna, Michele Proietti, Xin Du, Bodo Grimbacher

**Affiliations:** ^1^ Institute for Immunodeficiency, Center for Chronic Immunodeficiency, University Medical Center Freiburg, Freiburg, Germany; ^2^ Department of Hematology, Shenzhen Second People’s Hospital, The First Affiliated Hospital of Shenzhen University, Shenzhen, China; ^3^ Department of Biotechnology and Biosciences, University of Milano Bicocca, Milan, Italy; ^4^ Department of Microbiology, Immunology and Infectious Diseases, College of Medicine and Health Sciences, Arabian Gulf University, Manama, Bahrain; ^5^ Laboratory of Transmission, Control and Immunobiology of Infection, Pasteur Institute of Tunis, University Tunis El Manar, Tunis, Tunisia; ^6^ Department of Rheumatology and Clinical Immunology, Hannover Medical School, Hannover, Germany; ^7^ Resolving Infection Susceptibility (RESIST)—Cluster of Excellence 2155, Hannover Medical School, Hannover, Germany; ^8^ German Center for Infection Research (DZIF), Satellite Center Freiburg, Freiburg, Germany; ^9^ CIBSS—Centre for Integrative Biological Signalling Studies, Albert-Ludwigs University, Freiburg, Germany; ^10^ Resolving Infection Susceptibility (RESIST)—Cluster of Excellence 2155 to Hannover Medical School, Satellite Center Freiburg, Freiburg, Germany

**Keywords:** PGM3 insufficiency, infections, CD4+ T cells, UDP-GlcNAc, glycosylation

## Abstract

**Background:**

Hypomorphic mutations in the *phosphoacetylglucosamine mutase 3* (*PGM3*) gene cause a glycosylation disorder that leads to immunodeficiency. It is often associated with recurrent infections and atopy. The exact etiology of this condition remains unclear.

**Objective:**

This study aimed to characterize the phenotypes and immunological features associated with PGM3 insufficiency and investigate potential disease mechanisms.

**Methods:**

A systematic review of 44 published cases of PGM3 variants was performed, followed by T-cell phenotyping of two patients with PGM3 variants. A genotype-phenotypic severity study was conducted by comparing the residual PGM3 expression of the 12 reconstituted variants in human B cells. A PGM3 inhibitor was used to assess its effect on CD4+ T cell proliferation and differentiation.

**Results:**

Patients with PGM3 variants frequently presented with recurrent infections and atopy, accompanied by reduced naïve CD4+ T cell counts. A genotype–phenotype study showed that low levels of residual PGM3 expression are correlated with disease severity. Notably, inhibition of PGM3 activity impaired TCR-mediated CD4+ T cell proliferation and the synthesis of UDP-GlcNAc, complex N-glycans, O-GlcNAc, glycolytic stress, and mitochondrial respiration during proliferation in a dose-dependent manner. Partial loss of PGM3 activity was observed to preferentially enhance Th1 and Th2 differentiation, while attenuating Th17 and Treg differentiation, consistent with clinical observations.

**Conclusion:**

PGM3 is a critical regulator of CD4+ T-cell proliferation and differentiation. These findings provide new insights into the diverse clinical manifestations and therapeutic development of PGM3 deficiency.

## Introduction

Phosphoacetylglucosamine mutase (PGM3) is a pivotal enzyme in the nutrient-dependent hexosamine biosynthesis pathway (HBP) (1). In this pathway, PGM3 catalyzes the conversion of *N*-acetylglucosamine 6-phosphate (GlcNAc-6-P) into *N*-acetylglucosamine 1-phosphate (GlcNAc-1-P), which is an essential step in the generation of uridine diphosphate N-acetylglucosamine (UDP-GlcNAc). As a pivotal nucleotide precursor, UDP-GlcNAc facilitates various cellular glycosylation processes including N-linked glycosylation and O-GlcNAcylation of proteins. Studies in murine models have demonstrated the role of Pgm3 in regulating UDP-GlcNAc biosynthesis *in vivo*: Pgm3 deficiency manifests as embryonic lethality, whereas hypomorphic *PGM3* alleles result in aberrant glycosylation with systemic abnormalities and leukocyte reduction (1). Biallelic *PGM3* mutations in humans result in a range of immunological disease phenotypes, from Hyper IgE syndrome (HIES)-like phenotypes to combined immunodeficiency (CID) presentations and even severe combined immune deficiency (SCID)-like diseases with lymphopenia, syndromic features such as skeletal abnormalities, and developmental delay (2–15). However, an increasing number of case reports indicate that most affected individuals, except for those with prenatal onset, have increased susceptibility to infections accompanied by a reduction in CD4+ T cells. This finding suggests that PGM3 plays a significant role in the development of CD4+ T cells.

A quantitative loss of circulating CD4+ T lymphocytes and a reduction in mitogen-induced proliferative capacity are common among individuals with *PGM3* mutations. The role of PGM3 in CD4+ T cell development and homeostasis remains poorly understood. The downstream consequence of insufficient PGM3 activity is reduced UDP-GlcNAc synthesis, which directly affects both N-glycosylation and O-GlcNAcylation. The reduction in complex N-linked glycans has been observed in both murine models and in patient-derived cells with hypomorphic *PGM3* alleles (1, 3, 5, 16). While there is evidence from murine studies indicating reduced O-GlcNAcylation, there is a lack of research on this post-translational modification in humans. Emerging evidence suggests that O-GlcNAcylation plays an indispensable role in modulating T-cell activation, proliferation, and subset differentiation (17–19). Based on these findings, we postulated that insufficient PGM3 impairs T cell development by reducing UDP-GlcNAc synthesis, complex N-glycans, and O-GlcNAcylation protein post-translational modifications.

In T cells, glycolysis provides the energy required to maintain rapid proliferation and differentiation. An intermediate metabolite of the glycolytic pathway, fructose-6-phosphate, is involved in the PGM3-mediated hexosamine biosynthesis pathway. Therefore, it is relevant to ask whether insufficient PGM3 enzyme activity may have an impact on the overall glucose flux through changes in substrate availability or downstream production for the PGM3 reaction.

**Figure 1:**
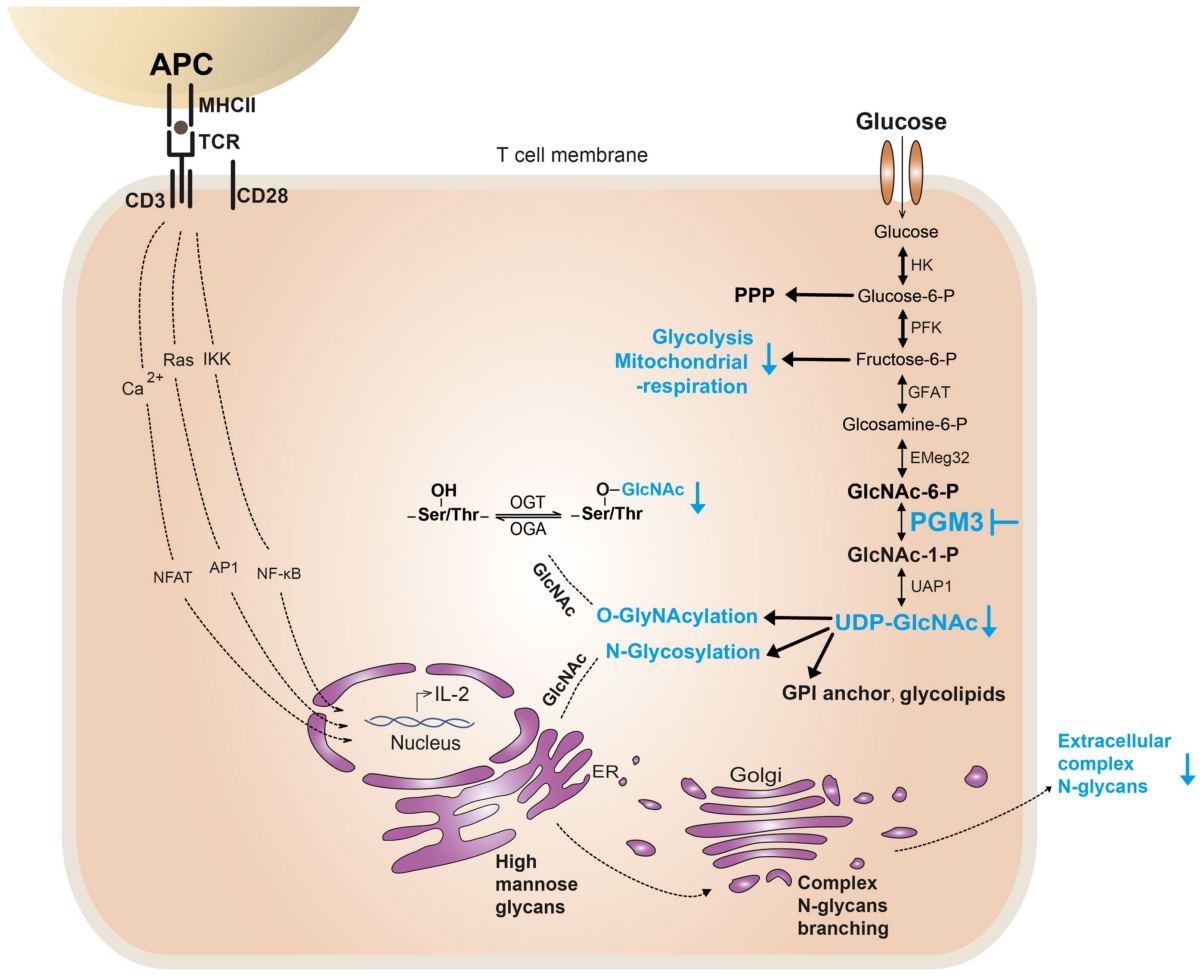
Potential mechanisms of PGM3 insufficiency causing T-cell defect. In T cells, PGM3 inhibition (in blue) reduces the synthesis of UDP-GlcNAc, which further affects the synthesis and protein modification of complex N-glycans and O-GlcNAc. Furthermore, PGM3 inhibition diminished metabolic pathways, including glycolysis and mitochondrial respiration, during TCR-induced cell proliferation. PPP, pentose phosphate pathway.

In this study, we characterized the clinical and immunological features of patients with hypomorphic *PGM3* mutations. We presented a genotype–phenotype correlation and demonstrated that inhibition of PGM3 activity markedly impaired TCR-induced CD4+T cell proliferation through a reduction in the synthesis of UDP-GlcNAc, complex N-glycans, and O-GlcNAcylation, as well as metabolic pathways, including glycolysis and mitochondrial respiration (**Figure 1**). We also showed that PGM3 activity modulates the fate of CD4+ T cell subsets, enhancing T helper 1 (Th1) and Th2 polarization while attenuating Th17 and regulatory T cells (Tregs). These findings provide new insights into the role of insufficient PGM3 activity and HBP in T-cell defects.

## Methods

### Systematic review

A review of published cases of PGM3 variants in humans from 2014 to 2023 was conducted. The cases included 44 affected individuals and were analyzed to summarize their clinical manifestations, immunophenotyping, genetic mutations, therapeutic approaches, and survival outcomes. Subsequently, Kaplan–Meier analysis was performed using GraphPad Prism 9 to determine the overall probability of survival for patients with PGM3 deficiency over time.

### Samples and PGM3 inhibitor

Human samples were obtained in accordance with the ethical guidelines of the University of Freiburg (ethical approval number 302/13_171483) after obtaining informed consent from all the study participants. The human B cell line BJAB was previously provided by Professor Eibel of the Institute of Immunology in Freiburg and was subsequently cryopreserved in our laboratory. The PGM3 inhibitor FR054 was synthesized by Prof. Ferdinando Chiaradonna’s laboratory (20, 21). Cells were treated with or without PGM3 inhibitor at the indicated concentrations for 72 h or 96 h and used for further analysis.

### Cells and culture conditions

Human BJAB cells were cultured in RPMI1640 supplemented with 10% fetal calf serum (FCS) and 1% penicillin–streptomycin (100 U/ml). HEK293T cells were cultured in DMEM medium supplemented with 10% FCS and 1% penicillin–streptomycin (100 U/ml). Primary human T cells were isolated from healthy donors and maintained in penicillin-streptomycin containing X-VIVO20 medium supplemented with 10% FCS, with or without the cytokines described to induce T cell polarization. All the cells were incubated at 37°C in a humidified atmosphere containing 5% CO_2_.

### Generation of PGM3-deficient and PGM3-reconstituted BJAB cells

To generate PGM3 knockout BJAB cells, single guide RNAs (sgRNAs) targeting the PGM3 sequence were cloned into the LentiCas9-GFP vector. The plasmid was then co-transfected with psPAX2 and pMD2.G packaging plasmids into HEK293T cells at a 4:3:1 ratio using XtremeGene HP transfection. After 24 h and 48 h, lentiviral particles were harvested, filtered (40 µM), and used to transduce BJAB cells by spinoculating at 2,000 rpm for 2 h with 4 μg/ml polybrene. The cells were sorted and diluted to a concentration of 1 cell/400 µl, and 200 µl of the solution was plated in each well of a 96-well plate. The expanded clones were evaluated for PGM3 knockout efficiency by Western blot analysis. Two pairs of sgRNA sequences (5’-3’) targeting *PGM3* were designed: (1) sense CACCGGACTGCTGGATTTCGAACGA and antisense AAACTCGTTCGAAATCC-AGCAGTC; (2) sense CACCGTATGGAAAGGC-AACTATAGA and antisense AAACTCTATAGTTGCCTTTCCATA.

To reconstitute wild-type and mutant PGM3, site-directed mutagenesis using two-step PCR introduced variants into the 542aa PGM3 cDNA sequence. This was performed using the pMXs-IRES-GFP-PGM3 plasmid as a template and the primers listed in **Table 1**. The purified PCR products were subjected to digestion and ligation using a linearized pMXs-IRES-mCherry retroviral vector as the recipient plasmid. The plasmid, together with the pCL-ampho packaging plasmid, was co-transfected at a 1:1 ratio into HEK293T cells using XtremeGene HP. The resulting retrovirus was harvested from the supernatant at 24-hour and 48-hour post-transfection. The virus was used to spinoculate PGM3-deficient BJAB cells with 4 μg/ml polybrene at 2,000 rpm for 2 h. After 7 days of incubation, mCherry-positive cells were sorted, expanded, and subjected to evaluation and assay for PGM3 expression.

**Table 1:** Primers used for introducing variants into wild-type PGM3 cDNA sequence.

No.	Mutants	Protein effects	Primers
1	c.715G-C	D239H	Fw: GTTCAGCTGTTTAATCATGGGTCCAAGGGCAAACTC Rv: GAGTTTGCCCTTGGACCCATGATTAAACAGCTGAAC
2	c.1135T-C	F379L	Fw: CATGGCACTGCACTGCTTAGTACAGCTGTTGAAATG Rv: CATTTCAACAGCTGTACTAAGCAGTGCAGTGCCATG
3	c.965 T-C	I322T	Fw: GGAGATTGGAGAAAGTTTGAATACTGGTGTTGTACAAACTGC Rv: GCAGTTTGTACAACACCAGTATTCAAACTTTCTCCAATCTCC
4	c.1018_1020del	E340del	Fw: GTTCAACACGGTATCTTGAAGTTATGAAGGTACCTGTC Rv: GACAGGTACCTTCATAACTTCAAGATACCGTGTTGAAC
5	c.737A-G	N246S	Fw: GGGTCCAAGGGCAAACTCAGTCATTTATGTGGAGCTGAC Rv: GTCAGCTCCACATAAATGACTGAGTTTGCCCTTGGACCC
6	c.737dupA	N246Kfs	Fw: GGGTCCAAGGGCAAACTCAAATCATTTATGTGGAGCTGAC Rv: GTCAGCTCCACATAAATGATTTGAGTTTGCCCTTGGACCC
7	c.975T-G	D325E	Fw: CACTTTCATCTCATAGAGGGAGACAAGATAGCAACG Rv: CGTTGCTATCTTGTCTCCCTCTATGAGATGAAAGTG
8	c.1585G-C	E529Q	Fw: CGGCCCTCTGGTACACAAGATGTCGTCCGAGTATATGC Rv: GCATATACTCGGACGACATCTTGTGTACCAGAGGGCCG
9	c.1438_1442del	L480fs	Fw: CAGATCTTCCAAACAGACAAAGTTCAGGTTGCAGAC Rv: GTCTGCAACCTGAACTTTGTCTGTTTGGAAGATCTG
10	c.248T-C	L83S	Fw: CCTTTGGGTGAAATGTCGGCACCATCCTGGGAG Rv: CTCCCAGGATGGTGCCGACATTTCACCCAAAGG
11	c.1504G-T	D502Y	Fw: CCCTCTGGTACAGAATATGTCGTCCGAGTATATGCAG Rv: CTGCATATACTCGGACGACATATTCTGTACCAGAGGG
12	c.1352A>G	Q451R	Fw: CAGATCTTCCAAACAGACGACTTAAAGTTCAGGTTGCAGAC Rv: GTCTGCAACCTGAACTTTAAGTCGTCTGTTTGGAAGATCTG
13	wt	WT	Fw: GATCGGATCCGCCACCATGGATTTAGGTGCTATTAC Rv: GATCGAATTCTCAGAAACCTGGTTGGGGCCTTTCTCC

### Western blot

The expression of PGM3 protein in the cell lysates was quantified by Western blotting. Cells were lysed in cold RIPA lysis buffer (50 mM Tris–HCl pH 8.0, 150 mM NaCl, 1% SDS, 0.5% sodium deoxycholate, and 1× complete protease inhibitor cocktail) for 20 min at 4°C. The protein concentration in the cell lysates was determined using a Pierce BCA protein assay kit according to the manufacturer’s instructions. The results were calculated using GraphPad Prism 9 software. Protein samples (50 µg) were resolved by SDS-PAGE and transferred to a nitrocellulose membrane. The membrane was then incubated overnight with anti-PGM3 antibody (Sigma). Protein expression levels on the Western blots were quantified by densitometry analysis using the ImageJ software. The expression of tubulin or GAPDH was used to quantify protein loading.

### Naïve CD4^+^ T cell isolation

Primary human CD4^+^ T cells were isolated from peripheral blood mononuclear cells (PBMCs) of healthy donors using a negative selection kit (Miltenyi Biotec). Naïve CD4+ T cells were stained with fluorochrome-conjugated antibodies and purified by sorting CD4^+^CD25^−^CCR7^+^CD45RA^+^ cells.

### CD4+ T cell proliferation and differentiation

To assess proliferation, naive T cells were labeled with CellTrace Violet dye (1:1,000) and cultured in 96-well plates precoated with anti-CD3 (OKT3, 2 μg/ml) and soluble anti-CD28 (CD28.2, 1 μg/ml) antibodies. At 72 h post-stimulation, cells were harvested and analyzed for complex N-glycans using PHA-L lectin staining, O-GlcNAc by immunostaining, glycolysis, and mitochondrial respiration using a Seahorse instrument. At 5 days post-stimulation, cell division was evaluated by flow cytometry to determine the dilution of CellTrace Violet, which serves as an indicator of cell division.

For cell polarization, naïve CD4^+^ T cells were activated on anti-CD3 antibody-coated plates (2 μg/ml) with soluble anti-CD28 antibodies (2 μg/ml) and the following cytokines: IL-12 (25 ng/ml) for Th1, IL-4 (40 ng/ml) and IL-2 (100 U/ml) for Th2, TGFβ (15 μg/ml) and IL-2 (100 U/ml) for Treg, and IL-1β (12.5 ng/ml), IL-6 (25 ng/ml), IL-21 (25 ng/ml), IL-23 (12.5 ng/ml) and TGFβ (5 ng/ml) for Th17 subset differentiation. After 5–7 days, the cells were re-stimulated with PMA (500 ng/ml) and ionomycin (100 ng/ml) in the presence of brefeldin A (10 μg/ml) for 4 h, and the expression of IFN-γ, IL-4, IL-17,and FoxP3 in Tregs was measured by flow cytometry. The data were acquired on a Fortessa flow cytometer, analyzed using FlowJo VX software, and subsequently quantified using GraphPad Prism.

### UDP-GlcNAc quantification by HPLC

CD4+ T cells were isolated and metabolically quenched with cold water/methanol/chloroform/(1:1:1). After incubation and centrifugation, the polar phase was purified by centrifugal filtration (Amicon, Merck) and evaporated under vacuum for storage at −20°C until analysis. UDP-GlcNAc levels in the samples were quantified by HPLC (Jasco Europe, Italy) using a C18 column (Kinetex, 4.6 mm × 250 mm i.d., 5 um particle size, 100 Å pore size, Phenomenex) with a guard column using a method adapted from a previous study (22). The working system was operated at 40°C and a flow rate of 1 mL/min. Samples (20 μl) were injected, and compounds were separated over a 75 min gradient using buffer A (100 mM potassium phosphate + 8 mM tetrabutylammonium hydrogen sulfate) and buffer B (100% acetonitrile) as follows: 0 min–12 min 100% A; 12 min–34 min 0%–30% B; 34 min–40 min 30% B; 40 min–50 min 30%–0% B; 50 min–75 min 100% A. UDP-GlcNAc was detected at 254 nm and quantified by comparison with standard curves. To maintain the performance, the column was washed after each sample set with solutions for 30 min each: 50% acetonitrile-50% phase A without ion-pair agents to remove retained analytes, 20% acetonitrile-80% water to remove salts, and 80% acetonitrile-20% water for storage.

### Statistical analysis

Statistical analyses were performed using GraphPad Prism 9. Student’s t-test was used to ascertain the significance of the difference between two groups, whereas one-way analysis of variance (ANOVA) was used to evaluate the disparity among the means of three or more independent groups. A p-value of less than 0.05 was considered to indicate statistical significance. The level of statistical significance is indicated as follows: *p <0.05, **p <0.01, ***p <0.001, and ****p <0.0001.

## Results

### Clinical features

**Figure 2:**
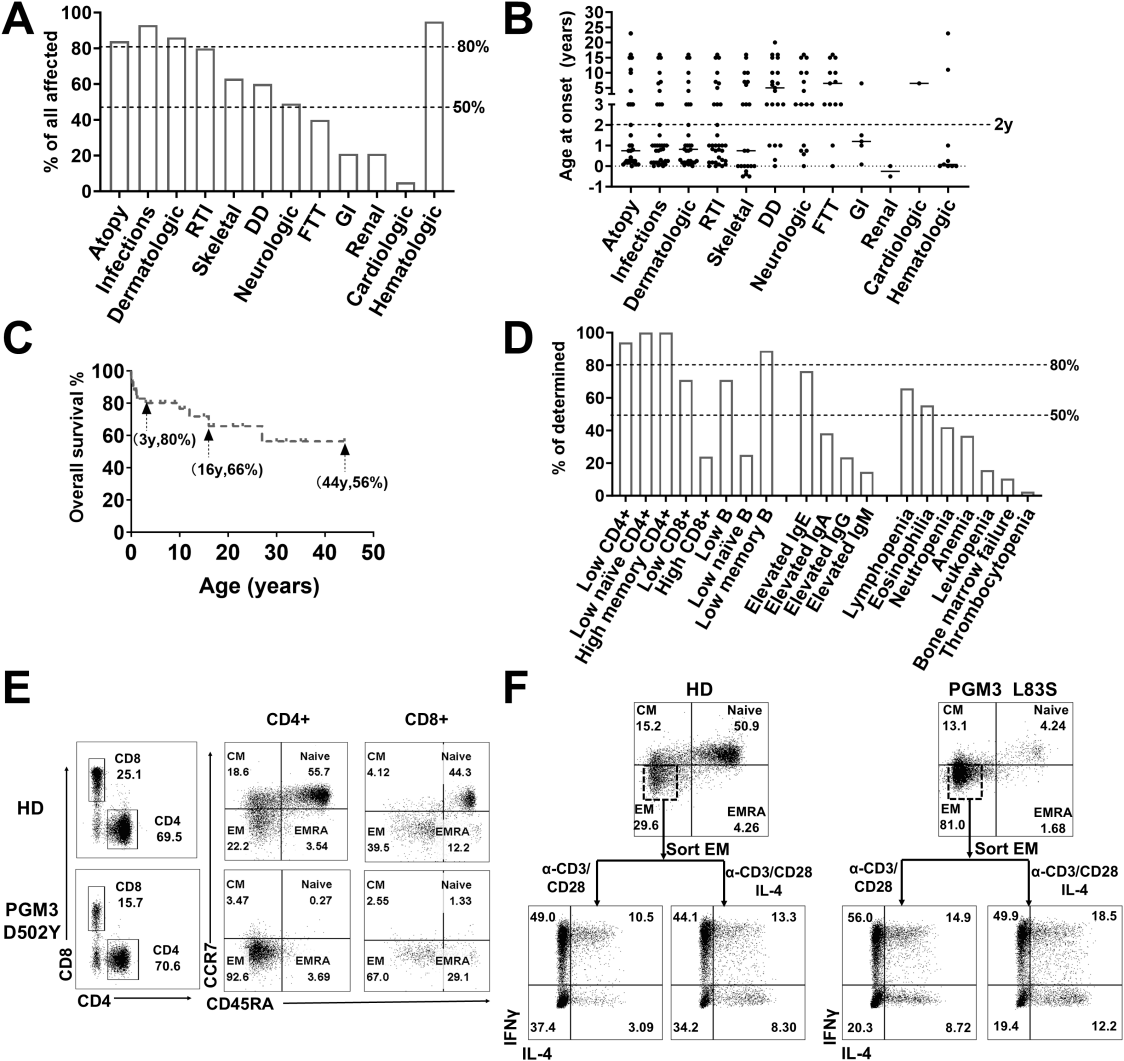
Clinical and immunological characterization of PGM3 insufficiency. **(A)** Clinical manifestations and **(B)** age of onset in patients with *PGM3* mutations. **(C)** Overall survival of patients with PGM3 insufficiency. **(D)** Summary of immunological features of patients with PGM3 mutations. **(E)** T cell phenotyping in family controls and one patient with PGM3 D502Y. **(F)** Differentiation of Th1 and Th2 subsets from restimulated effector memory (EM) cells derived from one patient identified with PGM3 L83S and one healthy control. RTI, respiratory tract infections; DD, developmental delay; FTT, failure to thrive; GI, gastrointestinal tract infections. EM, effector memory cells; CM, central memory cells; EMRA, effector memory cells re-expressing CD45RA.

A review of 44 cases from 21 families with biallelic *PGM3* mutations during the period 2014–2023 stratified the phenotypes as hyper IgE syndrome-like [HIES, 26 patients (3, 5, 8)], combined immunodeficiencies [CID, seven patients (2, 6, 10, 12)], and severe combined immunodeficiencies [SCID, 11 patients (4, 7, 13, 15)] (**Table 2**). Characteristic features included the presence of infections (93%) and atopy (84%) at a median age of 1 year (**Figures 2A, B**). Recurrent respiratory tract infections occurred in 39/42 patients. The most common diagnoses were pneumonia (n = 32), otitis media (n = 21), and bronchiectasis (n = 13). Cutaneous infections are also common, typified by abscesses of the head, neck, and axilla. Eight patients had infections such as gastroenteritis, osteomyelitis, esophagitis, septicemia, or encephalitis (3, 5, 6, 8, 10). Bacterial pathogens were predominant (29/42), including *Staphylococcus aureus, Pseudomonas aeruginosa*, and *Proteus mirabilis*. Eighteen patients had viral infections (VZV, EBV, RSV, etc.), and 13 had candidiasis. Marked eczematous eruptions including eczema, asthma, and multiple allergies, occurred in 37 patients between early infancy and childhood. Nonimmunological features were frequently observed. These include skeletal defects *in utero* to childhood (61%), neurological disorders (48%), developmental delay (61%), and failure to thrive (40%). Other multisystem effects included renal, cardiac, and gastrointestinal involvement (23%). Of note, eight cases had prominent skeletal malformations *in utero* or at birth (4, 9, 15). Taken together, these clinical manifestations revealed a profound, early onset immunological disorder associated with multi-organ disease with a wide range of ages of onset (**Figures 2A, B**).

**Table 2:** Genetic mutations, clinical diagnosis, and outcomes of PGM3 insufficiency.

No.	ID	PGM3 AA changes	Diagnosis	Outcomes
1^3,8^	A.V.12 (P1)	p. E340del	HIES	Alive (7 y)
2^3,8^	A.V.13 (P2)	p. E340del	HIES	Deceased (14 m)
3^3,8^	A.V.14 (P3)	p. E340del	HIES	Deceased (13 m)
4^3^	A.V.18	p. E340del	HIES	Alive (6 y)
5 ^8^	P4	p. E340del	HIES	Alive (4 y)
6 ^8^	P5	p. E340del	HIES	Alive (16 y)
7 ^8^	P6	p. E340del	HIES	Alive (7 y)
8 ^8^	P7	p. E340del	HIES	Alive (15 y)
9 ^8^	P8	p. E340del	HIES	Alive (11 y)
10 ^8^	P9	p. E340del	HIES	Alive (17 y)
11 ^8^	P10	p. E340del	HIES	Deceased
12 ^8^	P11	p. E340del	HIES	Deceased
13 ^8^	P12	p. E340del	HIES	Alive (3 y)
14^3^	B.V.6	p. L83S	HIES	Alive (3 y)
15^3^	B.V.7	p. L83S	HIES	Alive (36 y)
16^3^	D.IV.2	p. L83S	HIES	Alive (21 y)
17^3^	D.IV.5	p. L83S	HIES	Alive (11 y)
18^3^	C.IV.7	p. D502Y	HIES	Alive (15 y)
19^5^	I.1	p. [E529Q, L480Sfs*10]	HIES	Alive (35 y)
20^5^	I.2	p. [E529Q, L480Sfs*10]	HIES	Alive (32 y)
21^5^	I.3	p. [E529Q, L480Sfs*10]	HIES	Alive (30 y)
22^5^	I.4	p. [E529Q, L480Sfs*10]	HIES	Alive (30 y)
23^5^	I.5	p. [E529Q, L480Sfs*10]	HIES	Deceased (27 y)
24^5^	II.1	p. D325E	HIES	Alive (15 y)
25^5^	II.2	p. D325E	HIES	Alive (10 y)
26^5^	II.3	p. D325E	HIES	Alive (1 y)
27^7^	Patient	p. N246S	SCID	Deceased (1 m)
28^4^	A.II.2	p. N246S	SCID	Alive (6 y)
29^4^	B.II.4	p. [D239H];[0]	SCID	Alive (11 y)
30^4^	C.II.1	p. [N246Kfs*7]; [Q451R]	SCID	Deceased (7 m)
31^9^	Case1	p. F379L	SCID	Deceased (7 d)
32^9^	Case2	p. F379L	SCID	Deceased (6 d)
33^10^	Patient	Exon7 del	CID	Deceased (3 y)
34^2,6^	H.D	p. I322T	CID	Deceased (12 y)
35^2,6^	N.D	p. I322T	CID	Deceased (10 y)
36^2,6^	E.D	p. I322T	CID	Deceased (16 y)
37^2,6^	A.D	p. I322T	CID	Alive (44 y)
38^11^	Patient	p.[T335A; N482Mfs*4]	SCID	Alive (9 y)
39^15^	P1	R49T	SCID	Deceased (1.3 y)
40^15^	F1	M423T	SCID	Deceased (*in utero*)
41^15^	P2	M423T	SCID	Deceased (3 m)
42^12^	P1	p. [T464I]; [Q478X]	CID	Alive (23 y)
43^12^	P2	p. [T464I]; [Q478X]	CID	Alive (11 y)
44^13^	Infant	p. [I322T]; [R492X]	SCID	Alive (1.3 y)

*HIES, Hyper IgE syndrome; CID, combined immunodeficiency; SCID, severe combined immune deficiency.

The current treatment options are of limited benefit to patients with *PGM3* mutations. Of the 44 patients, 39 received conventional antimicrobial treatment and prophylaxis to control infections, with only incomplete efficacy. Administration of intravenous immunoglobulin (IVIG) yielded minimal benefit. After intensive care, 14 patients still died of overwhelming infections or organ failure at a median age of 2 years (range 5 days to 27 years) (2–5, 7–10, 15). Hematopoietic stem cell transplantation (HSCT) resulted in complete immunological reconstitution in three out of four cases (4, 11, 12). However, two recipients still had developmental delays but normal cognition after transplantation (4). The sole death occurred in an infant transplanted at 8 months due to severe complications (15). The overall survival rate was 80% at 3 years, decreasing to 66% and 56% at 16 years and 44 years, respectively (**Figure 2C**). These findings highlight the challenges in controlling infections and eczema with appropriate therapies and prophylaxis. Additionally, HSCT may pose risks related to underlying multiorgan diseases.

### Immunologic characterization

Hematological features were reported in 38 patients, including lymphopenia (65%), eosinophilia (54%), neutropenia (43%), anemia (35%), bone marrow failure (11%), and thrombocytopenia (3%) (**Figure 2D**). Elevated serum IgE levels were present in 25/32 (78%) patients (range, 650–141,300 IU/ml; median, 9,320 IU/ml; normal, <130 IU/ml). Other serum IgA, IgG, and IgM levels varied among the patients.

The most common documented immunological feature was a reduction in CD4+ T lymphocyte count, which was observed in 32 of the 34 patients (94%, range 4–913/ul, median 312/ul) (**Table 3**, **Figure 2D**). Notably, all nine patients who underwent further T cell phenotyping exhibited a reduction in naïve CD4+ T cells, accompanied by an increase in effector memory subsets. In addition, in the two patients with normal CD4+ T cell counts, this trend was observed. These results suggest that PGM3 deficiency leads to impaired CD4+ T cell production and enhanced differentiation. Deficits in CD8+ T cells were documented in 71% of cases, and high CD8+ T cell counts were observed in 20% of cases. B cells are also frequently affected (71%), with a reduction in memory subsets in most cases. Further functional characterization revealed defective T cell expansion following mitogenic stimulation by anti-CD3/CD28 antibodies, phytohemagglutinin (PHA), purified protein derivative (PPD), or tetanus antigens (TT) in 15 of 20 patients (75%). Two studies have reported a decrease in T-cell receptor excision circles (TRECS) associated with an increase in κ-deletion recombination excision circles (KRECs) (7, 10). Four studies evaluated T helper (Th) cell profiling towards hyperactive Th2 responses as evidenced by increased interleukin 4 (IL-4) production in contrast to variable Th1 (detected by IFNγ), Th17 (IL-17) and regulatory T (Treg) cell measurements (3, 5, 10, 12).

**Table 3:** T cell features in PGM3 insufficiency.

Pt No.	CD3+ T cells	CD4+ (359–1,565/ul)	Naïve CD4+	CD8+ (178–853/ul)	B cells (61–321/ul)	CD27+ mem B (12–68)
1^3,8^	2,450(2,120–2,680)	476(1,100–1,700)	unk.	1,994(635–1,235)	318(460–950)	unk.
2^3,8^	1,066(2,000–3,120)	328(1,270–2,300)	unk.	1,394(492–984)	760(574–1,520)	unk.
3^3,8^	1,435(2,000–3,120)	360(1,270–2,300)	unk.	1,030(492–984)	165(574–1,520)	unk.
4^3^	1,400(1,470–1,970)	381(740–1,220)	unk.	1,080(416–790)	224(370–870)	unk.
5 ^8^	606(1,400–3,700)	466(700–2,200)	unk.	236(490–1,300)	20(390–1,400)	unk.
6 ^8^	unk.	unk.	unk.	unk.	unk.	unk.
7 ^8^	unk.	unk.	unk.	unk.	unk.	unk.
8 ^8^	unk.	unk.	unk.	unk.	unk.	unk.
9 ^8^	1140(1,200–2,600)	337.5(650–1,500)	unk.	427(370–1,100)	75(270–860)	unk.
10 ^8^	unk.	unk.	unk.	unk.	unk.	unk.
11 ^8^	2,286(2,100–6,200)	595(1,300–3,400)	unk.	3,782(620–2,000)	453.6(720–2,600)	unk.
12 ^8^	204(1,400–3,700)	104(700–2,200)	unk.	105(490–1,300)	136(390–1,400)	unk.
13 ^8^	237.5(2,100–6,200)	157.5(1,300–3,400)	unk.	72.5(620–2,000)	77.5(720–2,600)	unk.
14^3^	1,100(2,120–2,680)	913(1,100–1,700)	12%	1,320(635–1,235)	630(460–950)	unk.
15^3^	2,490(1,470–1,970)	875(740–1,220)	11%	1,470(416–790)	1,470(370–870)	unk.
16^3^	Increased	unk.	unk.	unk.	normal	unk.
17^3^	Increased	unk.	unk.	unk.	normal	unk.
18^3^	670(456–580)	350(240–360)	2%	313(140–266)	7.6(100–205)	unk.
19^5^	low	128	unk.	129	253	7
20^5^	low	195–487	unk.	70–137	31–161	7
21^5^	low	93–151	unk.	11–19	100–123	2
22^5^	low	150–248	unk.	22–43	37–140	4
23^5^	unk.	unk.	unk.	unk.	unk.	unk.
24^5^	low	368–692	unk.	111–247	16–108	1
25^5^	normal	403–878	unk.	189–295	4–112	1
26^5^	low	324	unk.	263(500–2,000)	711(773–1,990)	11
27^7^	78	30	low	15	45	unk.
28^4^	low	low	unk.	low	low	unk.
29^4^	low	low	unk.	low	low	unk.
30^4^	low	low	unk.	low	low	unk.
31^9^	17	low	unk.	low	30	unk.
32^9^	low	low	unk.	low	low	unk.
33^10^	unk.	unk.	unk.	unk.	unk.	unk.
34^2,6^	unk.	unk.	unk.	unk.	unk.	unk.
35^2,6^	unk.	unk.	unk.	unk.	unk.	unk.
36^2,6^	370(780–2,010)	low	unk.	low	low	low
37^2,6^	730(2,100–6,200)	300(1,300–3,400)	unk.	430(620–2,000)	30(720–2,600)	unk.
38^11^	189(1,900–5,900)	low	low	18	414(610–2,600)	unk.
39^15^	11(2,500–5,500)	4(1600–4,000)	unk.	6	95(96–1,700)	unk.
40^15^	lymphopenia	low	unk.	low	unk.	unk.
41^15^	1,000	282	28.6%	low	normal	unk.
42^12^	500	low pronounced	15.8%	low	normal	unk.
43^12^	305	211	18%	47	398(636–2,829)	unk.
44^13^	600(900–4,500)	390	9%	180	180(160–2,000)	unk.

*unk., unknown.

Some studies have shown that patients with PGM3 insufficiency have recurrent infections associated with T lymphopenia, without skeletal or neurological involvement (11, 13). This indicated an important function of PGM3 in T cell development. Therefore, we tested T cell subsets from cells derived from two patients with PGM3 L83S and D502Y (3) mutations by flow cytometry. Our data showed extremely low percentages of naïve CD4+ T cell subsets (4.24% and 0.27%, respectively) in both patients compared with healthy controls (50.9%–55.7%) (**Figures 2E, F**). Furthermore, our *in vitro* assays on patient-derived effector memory cells revealed hyperactive Th1 and Th2 differentiation (**Figure 2F**). Unfortunately, we were unable to determine the levels of Th17 subsets due to limited blood resources. Consistent with the reviewed data, our results showed that depleted naive CD4+ T cell pools, in contrast to expanded effector memory subsets, may be a key feature of PGM3 insufficiency.

### Genotype and correlation to the phenotypic severity

**Figure 3:**
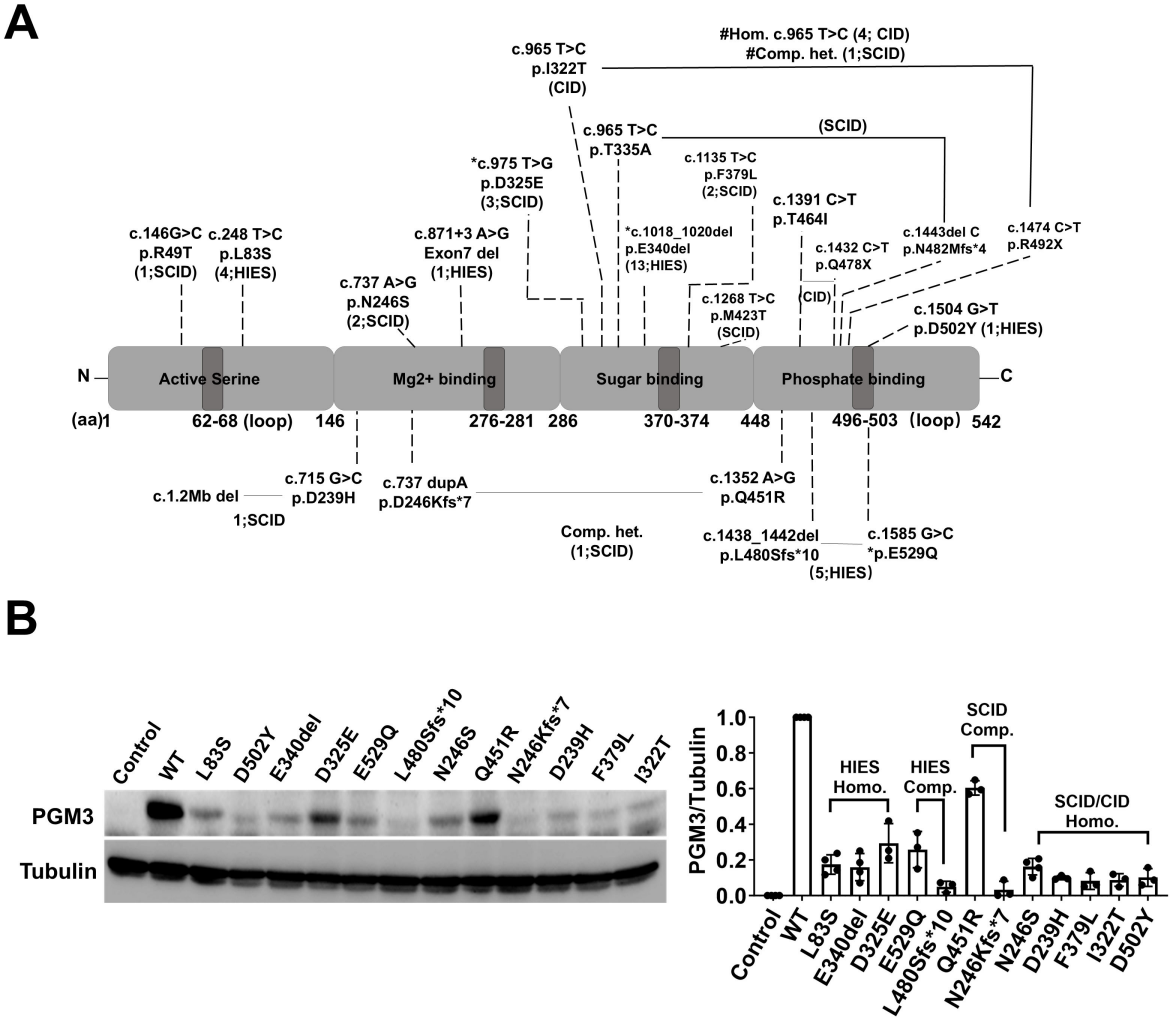
Genetic findings of PGM3 insufficiency and the corresponding expression of residual PGM3 protein. **(A)** Reported mutations and predicted amino acid changes are indicated by the broken lines. Two mutations linked by black lines indicate compound heterozygosity. The numbers in brackets are the number of affected individuals and associated diseases. *E529Q, L480Sfs*10, and D325E reported in the PGM3 isoform 570aa correspond to E501Q, L452Sfs*10, D297E in the PGM3 isoform with 542aa. # Homozygous I322T has been reported in four CID patients, whereas compound heterozygous p. [I322T; R492X] was reported in one SCID patient. **(B)** Expression of residual PGM3 in BJAB cells reconstituted with WT or mutated PGM3. PGM3-knockout cells were used as the control. The intensity of Western blot bands was quantified by Image J. The ratio of PGM3/Tubulin was calculated and normalized to that of WT PGM3. Data represent more than three independent experiments.

Molecular profiling revealed 21 distinct disease-causing mutations distributed across all four functional domains of the encoded PGM3 protein (**Figure 3A**). The D502Y and E529Q substitutions within the active site loop directly disrupt the phosphate binding function, whereas others are located between neighboring loops. The latter mutations are likely to alter the stabilizing effect of the loops or protein structure, directly or indirectly affecting protein stability or activity (4, 5). The resulting aberrant protein folding and instability appear to underlie reduced protein expression, enzymatic activity, and subsequent changes in protein glycosylation and show a robust genotype-phenotype correlation (3–6, 8, 10). Variants that cause minimal residual PGM3 expression, such as E340del and D502Y, correlate with more profoundly reduced synthesis of UDP-GlcNAc and more severe phenotypes presented in patients compared to L83S (3). The SCID phenotype or the fatal disease-associated variants, N246S and I322T are due to nearly undetectable protein levels and/or enzymatic activity (4, 6). Therefore, we hypothesized that the residual level of PGM3 expression and activity would indicate phenotypic severity.

To test the putative genotype-phenotype correlations, we used a CRISPR-based knockout and genetic reconstitution approach in human B cells (BJAB cells) to compare the residual levels of PGM3 expression resulting from 12 different variants (**Figure 3B**). This allowed for a side-by-side comparison of multiple disease-associated PGM3 alleles without the confounding effects of endogenous proteins. Western blot results showed that all 12 mutant PGM3 constructs were associated with reduced protein expression compared to the wild type (WT). The residual levels varied among individual mutations. Variants from SCID-CID cases (D239H, F379L, and I322T) showed less than 10% WT levels compared to 20%–30% in HIES-like cases (L83S, E340del, and D325E). However, the effects of the two variants reported in compound heterozygosity suggest partial additivity. In addition to reduced protein expression, variants that differentially affect enzymatic activity and subsequent UDP-GlcNAc synthesis and glycosylation are likely to further explain the complex genotype–phenotype relationships in this disorder.

### The role of PGM3 in CD4+ T cell proliferation and differentiation

**Figure 4:**
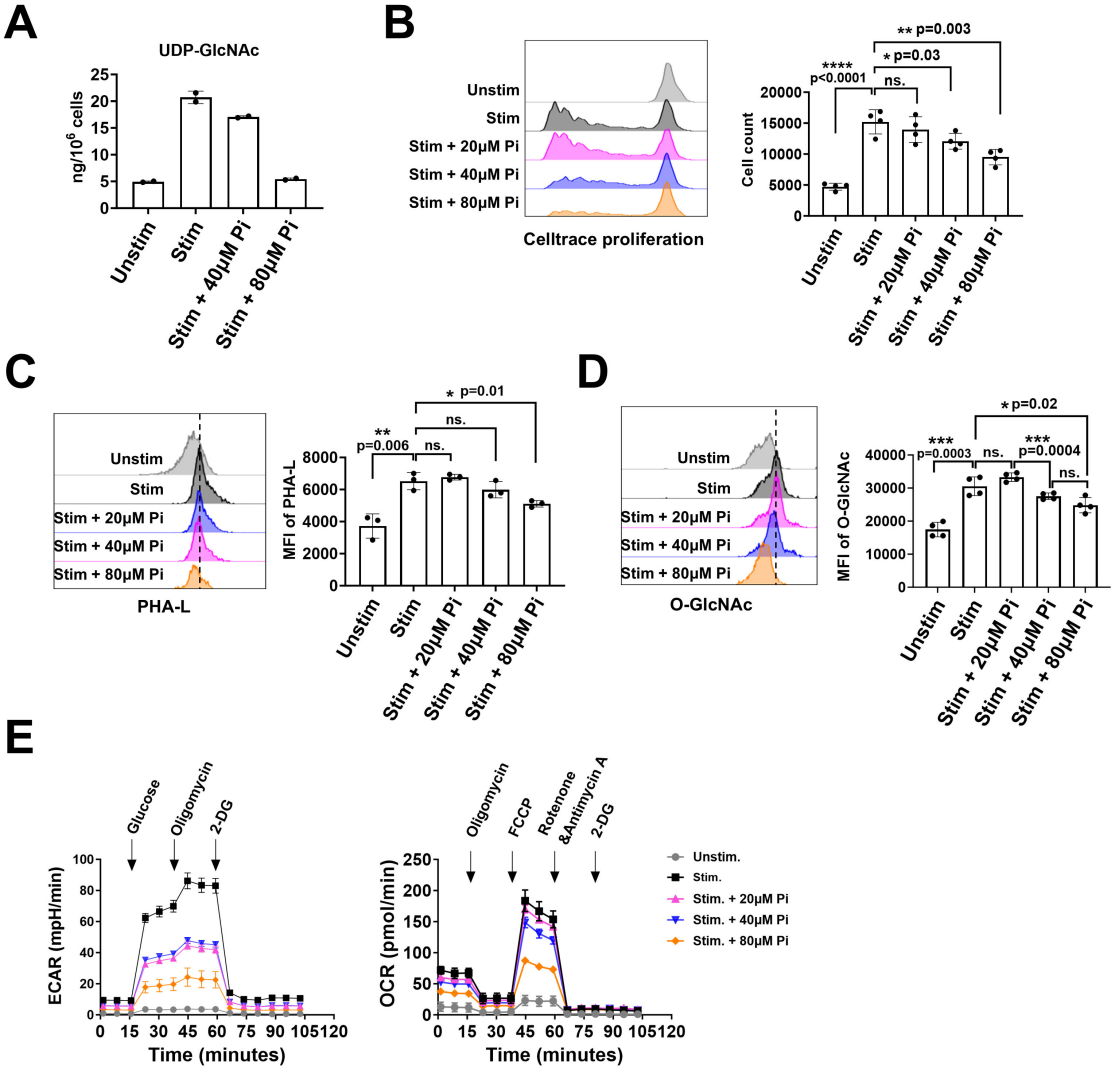
PGM3 inhibition in human T cells suppressed the synthesis of UDP-GlcNAc, protein glycosylation, and metabolic pathways in a dose-dependent manner. Inhibition of PGM3 activity in human primary T cells led to **(A)** reduced concentration of UDP-GlcNAc, **(B)** impaired cell proliferation, **(C)** reduced membrane complex N-glycans (reflected by the intensity of PHA-L staining), **(D)** reduced protein O-GlcNAcylation, **(E)** reduced glycolytic rate (left), and mitochondrial respiration (right) in stimulated CD4+ T cells. Pi, PGM3 inhibitor. Each experiment was performed in triplicate or tetraplicate. A repeat experiment was conducted to confirm these results. *p <0.05, **p <0.01, ***p <0.001, and ****p <0.0001. ns, no significance.

The predominant immunophenotype in patients with *PGM3* mutations is a marked decrease in CD4+ T cells *in vivo*, together with impaired cell differentiation in response to TCR-mediated stimulation *in vitro* (3, 5). This suggests an important role for PGM3 in TCR-mediated CD4+ T cell proliferation and differentiation. To investigate this, we used FR054, an inhibitor designed to inhibit PGM3 activity (20, 21), in cultures with human-derived CD4+ T cells. We first activated the cells using plate-bound anti-CD3/CD28 antibodies, quantified UDP-GlcNAc synthesis, and then analyzed cell growth, levels of complex N-glycans and O-GlcNAcylation, glycolysis, and mitochondrial respiration. Our data showed that 72 h of TCR-mediated cell stimulation increased UDP-GlcNAc levels fourfold compared to that in control cells. Treatment of cells with the PGM3 inhibitor reduced UDP-GlcNAc synthesis in a dose-dependent manner, as it caused 20% and 100% reductions at 40 μM and 80 μM, respectively, and almost blocked synthesis to the basal level observed prior to stimulation (**Figure 4A**). Notably, PGM3 inhibition impaired the TCR-induced cell proliferation. Cell count analysis showed a significant decrease in cell proliferation in parallel with a reduction in UDP-GlcNAc (**Figure 4B**). Similar decreasing trends were observed in the detection of complex N-glycans (indicated by PHA-L intensity, **Figure 4C**) on the cell surface and intracellular levels of O-GlcNAcylation, suggesting that even a small decrease in UDP-GlcNAc attenuated the synthesis of both complex N-glycans and O-GlcNAc during cell proliferation (**Figure 4D**). We also investigated the effects of PGM3 inhibition on metabolic pathways, specifically glycolysis and mitochondrial respiration, as reflected by changes in the extracellular acidification rate (ECAR) and oxygen consumption rate (OCR). As expected, 72 h of TCR stimulation significantly increased the glycolytic rate and mitochondrial respiration in control cells (**Figure 4E**). Treatment with the PGM3 inhibitor led to a dose-dependent reduction in both glycolysis and mitochondrial respiration (**Figure 4E**). Taken together, our results indicate that PGM3 inhibition impairs TCR-mediated cell proliferation through a reduction in UDP-GlcNAc levels, reduction in cell membrane complex N-glycans, and intracellular protein O-GlcNAcylation, associated with a significant decrease in cell energy and anabolic pathways.

**Figure 5:**
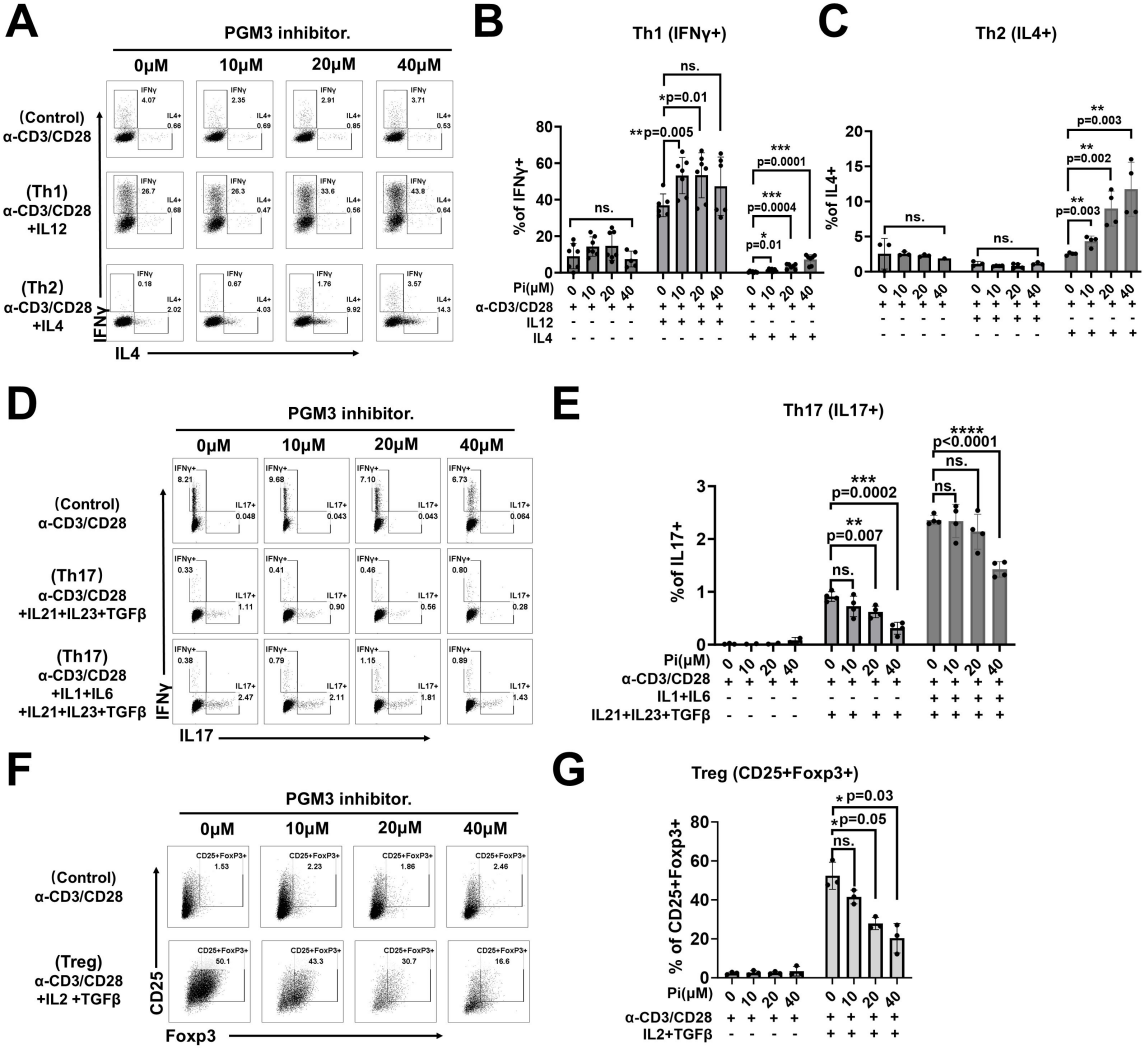
PGM3 inhibition in human naïve CD4+T cells affected T helper (Th) cell differentiation. Inhibition of PGM3 activity in human naïve CD4+T cells caused: **(A)** enhanced differentiation of Th1 (IFNγ+) and Th2 (IL4+) cells, **(B, C)** statistical analysis of Th1 and Th2 proliferation, **(D, E)** reduced Th17 (IL17+) proliferation, and **(F, G)** reduced Treg differentiation (CD25+Foxp3+) compared to controls. **(A, D, F)** indicate one of the three experiments corresponding to **(B, C, E, G)**, respectively. Each experiment was performed with triplicates or tetraplicates. A repeat experiment was conducted to confirm these results. *p <0.05, **p <0.01, ***p <0.001, and ****p <0.0001. ns, no significance.

Given that restimulated effector memory CD4+ T cells from a PGM3-mutant patient showed enhanced differentiation into Th1 and Th2 subsets, we investigated the role of PGM3 in modulating the differentiation of CD4+ T cell subsets. In *in vitro* studies, naïve CD4+ T cells were stimulated with anti-CD3/CD28 antibodies to induce polarization in the presence of subset-specific cytokines with or without low doses (≤40 μM) of the PGM3 inhibitor. Compared to vehicle controls, the presence of 10 μM–20 μM PGM3 inhibitor in culture significantly increased Th1 and Th2 differentiation, as measured by intracellular staining for IFNγ and IL-4, while 40 μM inhibitor further enhanced IL-4+ cells but not IFNγ+ cells (**Figures 5A–C**). In contrast, 20 μM PGM3 inhibitor significantly suppressed Th17 (IL-17+) and Treg (CD25+ FoxP3+) cell differentiation, whereas 40 μM caused a more profound reduction (**Figures 5D–G**). Taken together, we have shown that partial inhibition of PGM3 activity preferentially enhances Th1 and Th2 differentiation and attenuates Th17 and Treg cell differentiation.

In summary, these findings identified PGM3 as a novel regulator that affects CD4+ T cell proliferation and CD4+ T cell subset differentiation, providing new insights into heterogeneous clinical manifestations such as atopic diseases, variable infections, and autoimmune diseases, as listed in our review data.

## Discussion

Our study included extensive phenotypic and immunological analyses of 44 patients and functional evaluation of T cells to elucidate the pathogenic mechanisms underlying PGM3 insufficiency. The disease is characterized by infections, atopy, and multiorgan defects, often accompanied by the loss of naive CD4+ T cells and lymphopenia. In addition to residual PGM3 activity, our genotype–phenotype study revealed that residual PGM3 expression is an indicator of disease severity. At the cellular level, our *in vitro* studies identified PGM3 as a novel regulator of CD4+ T-cell proliferation and subset differentiation. This study provides new insights into disease manifestations and immunoprofiling of PGM3.

A genotype–phenotype correlation is predicted for PGM3 deficiency, as evidenced by the consistent association of the same mutations with comparable severity in unrelated patients, suggesting that different mutations allow for residual PGM3 expression and/or activity levels that influence clinical outcomes, ranging from moderate to severe immunodeficiency. A previous study by Stray-Pederson et al. demonstrated that extremely low PGM3 activity was associated with severe SCID phenotypes (4). In addition, our evaluation of 12 different PGM3 variants revealed variable reductions in the reconstituted protein expression levels in gene-edited human B cells. More severe SCID/fetal-associated variants consistently enabled less than 10% PGM3 expression, whereas 20%–30% levels were detected in cells expressing HIES-associated variants. This side-by-side comparison highlights the extremely low PGM3 expression (below 10% of normal) as a potential predictor of severe disease development. At the molecular level, residual PGM3 affects UDP-GlcNAc biosynthesis and subsequent glycosylation. Greater losses lead to more severe clinical and immunological manifestations. Consistent with this, our immunophenotyping study also revealed 0.27% naive CD4+ T cells in a patient with the D502Y variant compared with 4.2% in L83S. Clinically, the D502Y patient exhibited more profound CD4+ T-cell defects and severe features associated with lower levels of residual PGM3 expression. These findings confirm that the level of residual PGM3 expression and enzymatic activity contributes to disease severity in this disorder, both systemically and immunologically.

Recurrent infections paired with pronounced CD4+ T cell abnormalities were highly prevalent in PGM3-mutant patients, suggesting an important role of this enzyme in governing normal T cell development and function. At the molecular level, PGM3 inhibition impaired TCR-dependent proliferation and reduced the biosynthesis of UDP-GlcNAc, along with complex N-glycan branching and protein O-GlcNAcylation, as well as impaired glycolysis and mitochondrial respiration. Studies have demonstrated that N-glycosylation modifies numerous surface receptors that mediate cell signaling and intercellular interactions. Efficient activation and proliferation of naïve T cells requires co-stimulation of glycosylated TCR and CD28 (23). Thus, reduced PGM3 function is likely to disrupt the modification of TCR signaling proteins. Recent evidence also suggest that O-GlcNAcylated proteins such as c-Myc, NF-κB, and NFAT critically control T cell development (17, 24, 25). The expression and function of these proteins may also be affected by PGM3 deficiency. Interestingly, we observed dose-dependent impairment of glycolysis and mitochondrial respiration in PGM3-inhibited cells. Consistently, similar effects were observed in the two cancer cell models after treatment with the PGM3 inhibitor (unpublished data by Prof. Chiaradonna). We can argue that defective protein glycosylation and insufficient energy metabolism contribute to T-cell defects. Notably, numerous studies have highlighted the role of protein O-GlcNAcylation in the regulation of glycolytic enzyme expression and transporter activity that controls both glucose transport and mitochondrial metabolism (26–29), supporting the notion that PGM3 deficiency, which reduces UDP-GlcNAc and protein O-GlcNAcylation levels, could also affect metabolic pathways involved in T cell fate. Recent data have shown that upon activation, T cells increase glucose uptake, which facilitates increased oxidative phosphorylation and glycolysis to sustain cell growth and proliferation (30, 31). Therefore, it is therefore not surprising to hypothesize that PGM3 deficiency may have a significant effect on T cell metabolism. Whether and how PGM3 can reprogram the metabolism of highly proliferating cells, such as T cells, by promoting increased flux through the HBP or by modulating glycolysis, which is normally upregulated during proliferation, should be investigated further.

Moderate to severe atopic symptoms associated with elevated serum IgE levels were another clinical hallmark of PGM3. We initially hypothesized that aberrant IgE N-glycosylation results from reduced UDP-GlcNAc biosynthesis and complex N-glycan branching. However, our previous studies showed similar N-glycan profiling and *in vivo* stability of IgE proteins among samples derived from patients with elevated IgE-associated atopic dermatitis, HIES with LOF-STAT3, PGM3 insufficiency, and DOCK8 deficiency, compared to healthy individuals (unpublished data). Consistently, two glycoproteomic studies showed similar site-specific glycan profiles of IgE protein between a PGM3 mutant patient (p.E340del) and an atopic dermatitis patient (32) and between a healthy individual and a hyperimmune individual (33). These results suggested that PGM3 deficiency does not directly affect IgE glycosylation. However, in this study, immunophenotyping of memory CD4+ T cells from a patient with the PGM3 L83S variant revealed expanded Th2 subsets compared to healthy individuals, and increased Th2 polarization following TCR and IL-4 stimulation. Consistent with this, naïve CD4+ T cells treated with PGM3 inhibitor preferentially enhanced IL-4+ Th2 differentiation. Taken together, these findings implicate PGM3 reduction as a driver of uncontrolled Th2 responses, providing a causal explanation for the IgE dysregulation and high frequency of atopic diseases observed in patients.

In addition, our study also showed that partial loss of PGM3 activity spares cell replication but significantly enhances IFNγ+ Th1 and IL-4+ Th2 polarization, while reducing FOXP3+ Treg and IL-17+ Th17 polarization. This is consistent with immunophenotyping showing depleted naïve CD4+ T cell pools contrasted with expanded effector memory subsets in patients with L83S. This reduced pool of naïve CD4+ T cells may result from impaired thymic production or the release of naïve cells into the circulation, along with enhanced peripheral differentiation into effector lineages. Furthermore, the altered balance of CD4+ T cell subset polarization implies that normal PGM3 function is required to restrain overactive Th1/Th2 responses and promote sufficient Treg and Th17 subset development to maintain a proper balance between the inflammatory response and regulatory function. Hyperactive Th2 and reduced Treg differentiation are likely to contribute to elevated serum IgE levels, eosinophilia, and eczema in these patients, while reduced Th17 differentiation may also explain the susceptibility to bacterial and fungal infections. However, *in vitro* polarization assays only indicate differentiation capacity and may not reflect the final immune balance achieved *in vivo*, where complex regulatory interactions between CD4+ T cell subsets influence the final immunological outcome. Indeed, the predominance of atopy and infections over autoimmunity in patients suggests complex PGM3-dependent effects *in vivo*. Furthermore, the enzymatic role of PGM3 in generating UDP-GlcNAc and in broadly affecting the function of glycosylated proteins in numerous cell types may be involved in the pathogenesis of atopic and infectious diseases.

Ultimately, elucidation of pathogenic mechanisms aims to provide evidence to guide therapeutic development and improve long-term outcomes. This is particularly important because current conventional treatments have limited efficacy. Limited data suggest that HSCT is a potentially curative option for immunological symptoms but not for neurological impairment (7, 12, 15). A successful case report of an infant transplanted early after birth with reduced intensity conditioning survived with normal development, suggesting that early timing and appropriate regimens may improve success (13). In principle, the immediate consequence of PGM3 loss is reduced UDP-GlcNAc biosynthesis with downstream glycosylation defects underlying the associated consequences. Oral administration of GlcNAc has been shown to significantly increase the UDP-GlcNAc pool, glycoprotein production, and clinical improvement in pediatric inflammatory bowel disease (34, 35). Therefore, exploring the effects of GlcNAc treatment on PGM3 insufficiency may provide a promising and inexpensive strategy to correct metabolic deficiencies.

In conclusion, the extensive clinical and immunological profiling of patients carrying *PGM3* mutations highlights the prominent role of this enzyme in modulating glycosylation, particularly in processes governing immune function and development. Our integrated analyses established a genotype–phenotype correlation and provided novel causal insights into the cellular and molecular understanding of CD4+ T cell defects observed in this disorder. Further efforts are required to gain a deeper understanding of disease mechanisms, which will benefit the future development of therapeutic strategies for patients with PGM3 insufficiency.

## Data Availability

The original contributions presented in the study are included in the article/supplementary material. Further inquiries can be directed to the corresponding authors.
